# Molecular Detection of SARS-CoV-2 From Throat Swabs Performed With or Without Specimen Collection From the Tonsils: Protocol for a Multicenter Randomized Controlled Trial

**DOI:** 10.2196/47446

**Published:** 2024-06-12

**Authors:** Benedikte Hartvigsen, Kathrine Kronberg Jakobsen, Thomas Benfield, Niels Tobias Gredal, Annette Kjær Ersbøll, Mathias Waldemar Grønlund, Henning Bundgaard, Mikkel Porsborg Andersen, Nina Steenhard, Christian von Buchwald, Tobias Todsen

**Affiliations:** 1 Copenhagen Academy for Medical Education and Simulation Copenhagen Denmark; 2 Department of Otorhinolaryngology, Head and Neck Surgery and Audiology Rigshospitalet Copenhagen University Hospital Copenhagen Denmark; 3 Department of Clinical Medicine University of Copenhagen Copenhagen Denmark; 4 Department of Infectious Diseases Amager and Hvidovre Copenhagen University Hospital Hvidovre Denmark; 5 Copenhagen Emergency Medical Services University of Copenhagen Copenhagen Denmark; 6 National Institute of Public Health University of Southern Denmark Odense Denmark; 7 Department of Cardiology Rigshospitalet Copenhagen Denmark; 8 Cardiology Research Unit Nordsjællands Hospital Hillerød Denmark; 9 TestCenter Danmark Statens Serum Institut Copenhagen Denmark

**Keywords:** SARS-CoV-2, COVID-19, pandemic, oropharyngeal sampling, diagnostic accuracy, PCR, polymerase chain reaction, PCR analysis, swab, diagnostic, oropharyngeal, virology, testing, detection, molecular biology, microbiology, laboratory, palatine tonsil, COVID-19 detection, COVID-19 testing, tonsil, swabs, oropharyngeal swabs, oropharyngeal swab, nasal swab, nasal swabs, molecular detection, tool, diagnostic technique, diagnostic procedure, clinical laboratory techniques

## Abstract

**Background:**

Testing for SARS-CoV-2 is essential to provide early COVID-19 treatment for people at high risk of severe illness and to limit the spread of infection in society. Proper upper respiratory specimen collection is the most critical step in the diagnosis of the SARS-CoV-2 virus in public settings, and throat swabs were the preferred specimens used for mass testing in many countries during the COVID-19 pandemic. However, there is still a discussion about whether throat swabs have a high enough sensitivity for SARS-CoV-2 diagnostic testing, as previous studies have reported a large variability in the sensitivity from 52% to 100%. Many previous studies exploring the diagnostic accuracy of throat swabs lack a detailed description of the sampling technique, which makes it difficult to compare the different diagnostic accuracy results. Some studies perform a throat swab by only collecting specimens from the posterior oropharyngeal wall, while others also include a swab of the palatine tonsils for SARS-CoV-2 testing. However, studies suggest that the palatine tonsils could have a tissue tropism for SARS-CoV-2 that may improve the SARS-CoV-2 detection during sampling. This may explain the variation of sensitivity reported, but no clinical studies have yet explored the differences in sensitivity and patient discomfort whether the palatine tonsils are included during the throat swab or not.

**Objective:**

The objective of this study is to examine the sensitivity and patient discomfort of a throat swab including the palatine tonsils compared to only swabbing the posterior oropharyngeal wall in molecular testing for SARS-CoV-2.

**Methods:**

We will conduct a randomized controlled study to compare the molecular detection rate of SARS-CoV-2 by a throat swab performed from the posterior oropharyngeal wall and the palatine tonsils (intervention group) or the posterior oropharyngeal wall only (control group). Participants will be randomized in a 1:1 ratio. All participants fill out a baseline questionnaire upon enrollment in the trial, examining their reason for being tested, symptoms, and previous tonsillectomy. A follow-up questionnaire will be sent to participants to explore the development of symptoms after testing.

**Results:**

A total of 2315 participants were enrolled in this study between November 10, 2022, and December 22, 2022. The results from the follow-up questionnaire are expected to be completed at the beginning of 2024.

**Conclusions:**

This randomized clinical trial will provide us with information about whether throat swabs including specimens from the palatine tonsils will improve the diagnostic sensitivity for SARS-CoV-2 molecular detection. These results can, therefore, be used to improve future testing recommendations and provide additional information about tissue tropism for SARS-CoV-2.

**Trial Registration:**

ClinicalTrials.gov NCT05611203; https://clinicaltrials.gov/study/NCT05611203

**International Registered Report Identifier (IRRID):**

DERR1-10.2196/47446

## Introduction

### Background and Rationale

Testing for SARS-CoV-2 has been essential in limiting the spread of infection in society during the COVID-19 pandemic, where billions of tests have been performed worldwide [1]. Further, sensitive tests for SARS-CoV-2 are also important to provide early COVID-19 treatment for people at high risk of severe illness. Proper upper respiratory specimen collection is the most critical step in diagnosing COVID-19, and standard sample guidelines are recommended [2]. A throat swab, alone or combined with a nasal swab, is a frequently used sample method in many countries [3-5]. However, there is considerable variability in the reported diagnostic accuracy of throat swabs, with a 95% CI from 52% to 100% reported in systematic reviews [6,7]. Several studies have found that throat swabs have significantly lower sensitivity than other respiratory specimens [8,9] and the Infectious Diseases Society of America, therefore, does not recommend throat swabs for SARS-CoV-2 testing [10]. In contrast, an experimental study with volunteers inoculated with SARS-CoV-2 intranasally to assess virus kinetics found that SARS-CoV-2 molecular testing of throat swabs was positive before nasal swabs during the asymptomatic infectious stage [11]. Many of the previous studies performing throat swabs also lack a detailed description of the sampling technique, which makes it difficult to compare the different diagnostic accuracy results. Some studies perform a throat swab by only collecting specimens from the posterior oropharyngeal wall [12,13], while others also include a swab of the palatine tonsils for SARS-CoV-2 testing [14,15]. However, recent studies suggest that throat swabs, including the palatine tonsils, have a higher sensitivity for SARS-CoV-2 detection than nasal and nasopharyngeal swabs in asymptomatic and early infectious stages [16,17]. SARS-CoV-2 primarily uses the tissue-specific proteases TMPRSS2, TMPRSS4, and TMPRSS11D as cellular entry factors via angiotensin-converting enzyme 2 channels, which are richly expressed in the tonsil crypts [18,19]. This may explain why SARS-CoV-2 can be detected in throat swabs before nasal swabs, underlining timing as a crucial factor for test results [20]. Many factors may contribute to the variation of sensitivity reported, but no clinical studies have explored the clinical role of the palatine tonsils in diagnostic testing for SARS-CoV-2 in community settings. We will, therefore, conduct a randomized clinical trial to compare the diagnostic sensitivity of a throat swab with or without the palatine tonsils.

### Research Question

The question of this research is, in a cohort of individuals tested for COVID-19 at a public test center, what is the sensitivity of a throat swab including the palatine tonsils compared to only swabbing the posterior oropharyngeal wall in molecular detection of SARS-CoV-2?

## Methods

### Overview

We will conduct a randomized controlled trial to compare the molecular detection rate of SARS-CoV-2 by a throat swab performed from the posterior oropharyngeal wall and the palatine tonsils (intervention group) or the posterior oropharyngeal wall only (control group).

### Study Setting

Participants will be enrolled in a free public COVID-19 test center in Valby and Hillerød, Capital Region, Denmark starting from November 10, 2022, until December 22, 2023. All samples will be sent for molecular testing at Statens Serum Institut and data analysis will be performed at Rigshospitalet.

Only specially trained health care workers (HCWs) at Valby and Hillerød test center will collect specimens from the participants enrolled in the study. Before enrollment, all HWCs will receive training and a competency-based assessment of their skills [21] (see Multimedia Appendix 1 for details).

Follow-up data will be collected from study enrollment until 3 months post enrollment by an electronic questionnaire sent via REDCap (Vanderbilt University).

### Eligibility Criteria

Individuals with or without symptoms of upper respiratory tract infection, aged 18 years or older, who visit Valby or Hillerød test center for reverse transcriptase–polymerase chain reaction (RT-PCR) testing for SARS-CoV-2 will be offered participation in the study.

The exclusion criteria are individuals with a tracheostomy, laryngectomy, or prior oropharyngeal cancer surgery that would make the throat swab difficult. Further, individuals without a Danish civil registration number will be excluded from participating. Individuals who are not included in the study will have the standard oropharyngeal swab performed in the test center.

An individual will only be allowed to participate in the study once. If a participant is enrolled in the study more than once, only the first enrollment (or the first positive test case) will be included. The following test results will be excluded from the statistical analysis by the statistician who will remove any duplicates of the civil registration numbers.

### Randomization to Intervention and Control Group

**Figure 1:**
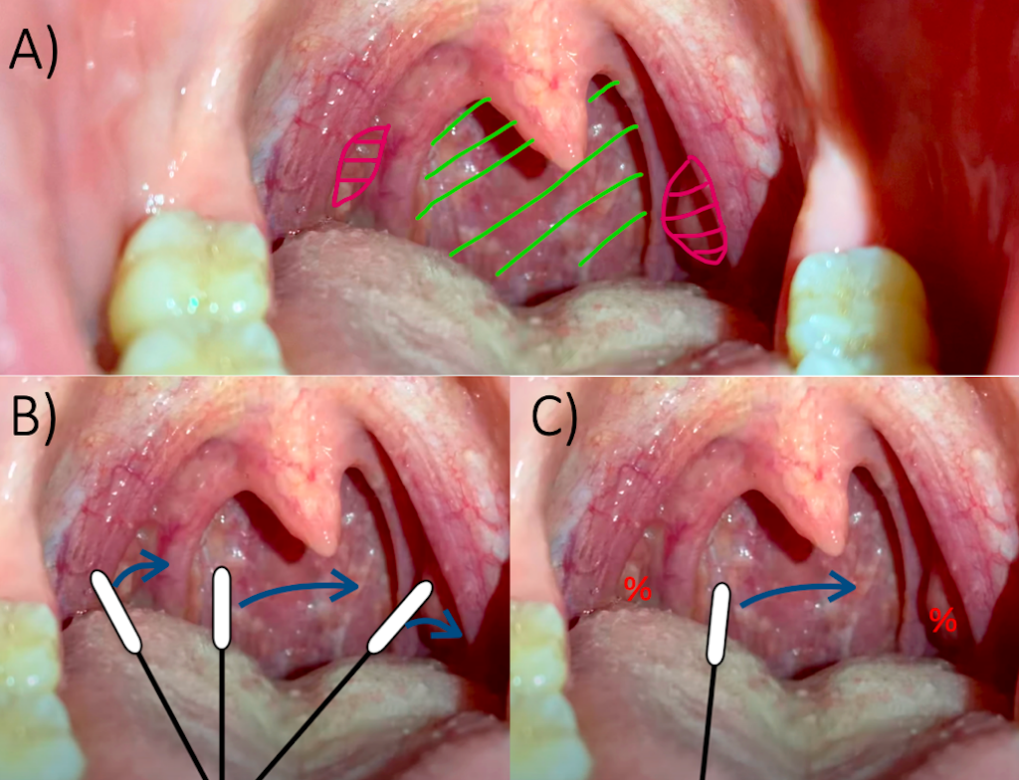
(A) Anatomic visualization of the posterior oropharyngeal wall (green) and palatine tonsils (pink). (B) Throat swab including the posterior oropharyngeal wall and palatine tonsils. (C) Throat swab including the posterior oropharyngeal wall but avoiding the palatine tonsils. %: no swabbing was performed from the palatine tonsils.

After enrollment, the participants will be randomized in a 1:1 ratio either having the throat swab performed including the posterior oropharyngeal wall and both palatine tonsils (intervention group) or only including the posterior oropharyngeal wall and avoiding the palatine tonsils (control group; see Figure 1).

**Figure 2:**
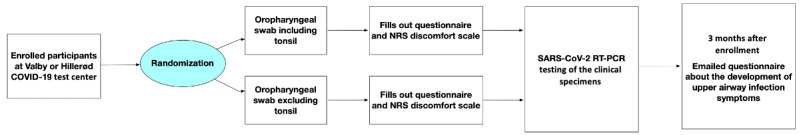
Study flowchart of the randomized controlled trial study design including volunteer participants from 2 public SARS-CoV-2 test centers in Copenhagen Municipality between November 2022 and March 2023. NRS: numerical rating scale; RT-PCR: reverse transcriptase-polymerase chain reaction.

A block randomization list was generated by author TT using the online randomization software Sealed Envelope [22] and afterward uploaded to REDCap. The randomization group will be disclosed in connection with trial registration in REDCap using the REDCap “randomize” function. The throat swab will afterward be performed by the trained HWCs with or without swabbing of the palatine tonsils depending on the randomization to either the intervention or control group, see Figure 2.

The swabs will be placed in separate dry tubes and sent to the Statens Serum Institute, Copenhagen, Denmark, for detection of SARS-CoV-2 by single-target RT-PCR. Samples with viral cycle threshold (Ct) values below 38 are considered positive, 38-40 inconclusive, and above 40 negative [23].

All participants are required to fill out a baseline survey on site about their reason for being tested, prior COVID-19 infections, vaccination status, symptoms, and if any prior tonsillectomy has been performed (Multimedia Appendix 1).

All HCWs participating in the data collection have been given a unique ID that will be registered for each throat swab performed in the study. Further, the HCW will also be asked to rate the Mallampati score of the participants included in the study (Multimedia Appendix 1). All data will be documented on site in a secure web database (REDCap; Multimedia Appendix 1).

### Outcomes

The primary outcome will be reported as SARS-CoV-2 RNA by RT-PCR test result (positive, negative, and inconclusive). The secondary outcome will be reported as (1) SARS-CoV-2 RT-PCR Ct value, (2) test discomfort on an 11-point numerical rating scale (NRS), (3) development of COVID-19 disease after testing, (4) SARS-CoV-2 detection rate for each HCW, and (5) Mallampati score of participants being tested.

### Sample Size

Based on a SARS-CoV-2 test positive proportion of 13% in the Danish capital region in the last week of October 2022, we expected the proportion of positive tests to increase to about 20% during the following study period [24]. The power calculation was based on an expected improvement in diagnostic accuracy of 25% by including the palatine tonsils in the throat swab compared to a sample without the tonsils. Using the ClinCalc [25] sample size calculator, we, therefore, estimated that a sample of 2188 participants would provide the trial with 80% power at a 5% significance level with an expected test-positive proportion of 20% equal to 438 individuals positive for SARS-CoV-2 among the participants tested. As we expect about 219 (10%) participants missing due to dropout and missing data, we aim to include 2407 participants in the study. All individuals entering the testing facility who meet the eligibility criteria are offered to participate in the study. We expect about 300-700 tests performed at both COVID-19 test centers each day and an assumed participation of 10%-30%. We, therefore, expect to complete the enrollment within 3 months.

### Statistical Analysis

A study participant is considered to have a SARS-CoV-2 infection if the throat swab is RT-PCR positive (reference standard). Participants with an inconclusive RT-PCR test result will be included in the analyses as a negative test result following an intention-to-diagnose approach [26]. Differences in the proportion of SARS-CoV-2–positive tests between the intervention and the control group will be compared using binary logistic regression using the test center as a fixed effect and a generalized estimating equation to adjust for clustering of data within the HCWs performing the sample (see the statistical analysis plan in Multimedia Appendix 1 for further details). The difference in SARS-CoV-2 detection rate between HCWs will also be reported separately to estimate the potential difference in sampling efficacy. The Ct values from positive RT-PCR samples and the NRS discomfort scores will be compared using a general linear model with mixed effects (Ct) and generalized estimating equation (GEE) models (NRS discomfort). The 95% CI will be presented. Categorical data will be summarized with numbers and percentages, while continuous data will be summarized by mean and SD. We will not perform tests of statistical significance for baseline characteristics. A significance level of 5% was applied. Statistical analysis software (version 9.4; SAS Institute) will be used for the statistical analyses.

### Planned Subgroup Analyses

We planned to do a sensitivity analysis using a lower Ct<30 for positive SARS-CoV-2 definition to explore the consequences of a higher test specificity for the SARS-CoV-2 detection rate between specimen types. Further, we plan to do subgroup analyses exploring the distribution of positive test results for participants stratified by symptoms, age, sex, tonsillectomy, chronic diseases or conditions, smokers, previous COVID-19 infection, vaccination status, prior tonsillectomy, and Mallampati score. To explore a potential bias from the distribution of the inconclusive test results, we excluded the inconclusive results in the subgroup analyses.

### Quality Assurance

All the HCWs, who will be including participants in this study, will be trained and have at least 3 months prior experience performing throat swabs. Further, they will complete a special standardized training and certification program taught by a board-certified otorhinolaryngologist to ensure all HCWs perform the same technique on the participants in the intervention and control group [21]. A 2-hour long training session will cover the theoretical and practical part of throat swab sample techniques and the study design with the elements (1) a walk-through of the practical study setup, how participants are enrolled, and how diagnostic interventions are performed; (2) theory on throat swabbing including upper airway anatomy, anatomical variations, and Mallampati score; and (3) practical exercises in throat swab technique with and without tonsils.

Checklists outlining the 2 different sampling techniques for throat swabs with and without palatine tonsils, respectively, will be used to assess the swab performance of all the HCWs by a board-certified otolaryngologist or specially trained nurse before study enrollment (see Multimedia Appendix 1). An on-site health care professional (NTG) will be in charge of internal daily quality assurance.

The test center in Denmark’s PCR laboratory at Statens Serum Institut, which performs the PCR analyses for this study, is ISO/IEC 17025 accredited.

### Follow-Up

The participants enrolled in the study will receive an online questionnaire 3 months after enrollment to follow up on the number and length of symptoms after they were tested (see Multimedia Appendix 1).

### Ethical Considerations

This trial was reported to the Regional Ethics Committee of the Capital Region of Denmark which considered it exempted from further processing (H-22022937). All participants received oral and written information (Multimedia Appendix 1) before signing a consent form (Multimedia Appendix 1) upon enrollment in the trial, and no financial compensation was made to participants in the study. All data have been collected, anonymized, analyzed, and stored in accordance with Danish General Data Protection Regulation legislation. This trial is approved by the data responsible institute in the Capital Region of Denmark (P-2022-803). The protocol was registered with the ClinicalTrials.gov database (NCT05611203). No generative artificial intelligence was used in any portion of the paper writing. Participation is voluntary and participants are required to provide oral and written consent to participate before entering the study (Multimedia Appendix 1).

## Results

A total of 2315 participants were enrolled in the study between November 10, 2022, and December 22, 2022. By March 22, 2023, follow-up data collection was completed. The research group is currently finalizing the statistical analyses and preparing the paper for publication aiming to publish primo 2024.

## Discussion

### Principal Findings

This randomized clinical trial will provide us with information about whether throat swabs including specimens from the palatine tonsils will improve the diagnostic sensitivity for SARS-CoV-2 molecular detection. These results can, therefore, be used to improve future testing recommendations, as well as it will provide additional information about the tissue tropism for SARS-CoV-2.

### Study Design

The scope of our study is to compare the diagnostic accuracy of 2 different techniques for throat swabs—with or without swabbing the palatine tonsils. We, therefore, aimed to conduct the study in a way that SARS-CoV-2 testing is currently performed in clinical practice with as little as possible of change. The HCW performing the throat swab was, therefore, instructed to do as usual and only focus on performing the swab including the posterior oropharyngeal wall and both palatine tonsils (intervention group) or only including the posterior oropharyngeal wall (control group), depending on randomization (see Figure 1). This setup has several strengths. First, the participant only needs 1 swab which is expected to make enrollment easier. Second, the HCW performing the test has to make only minimal changes to their usual routine. This ensures a high degree of familiarity with the test setting minimizing the risk of human error and optimizing time consumption. Finally, all samples in the study are handled the same way as any normal sample which ensures that our test analyses are comparable to those not participating in the study.

However, another study design where each participant was swabbed twice from the tonsil and posterior oropharyngeal wall, respectively, could have provided a more direct comparison of the presence of SARS-CoV-2 detection from each anatomical location. However, such a design would also have several drawbacks. First, recruiting participants would likely prove more difficult as throat swabs are associated with discomfort and more people would volunteer to participate if only a single swab was performed. Next, this setup would require considerably more time per participant, which would be difficult to prioritize in a busy test center. Finally, it would also double the cost of swabs and molecular tests, increasing the overall cost of the study substantially.

However, another study design where each participant was swabbed twice from the tonsil and posterior oropharyngeal wall, respectively, could have provided a more direct comparison of the presence of SARS-CoV-2 detection from each anatomical location. Furthermore, this way of testing is not comparable to the way tests are performed in the test centers, and results, therefore, would not be directly transferrable to clinical practice.

### Study Setting

We chose to enroll participants from public COVID-19 test centers, as it gave us the opportunity to enroll a high volume of participants in a standardized test setting with HCWs specially trained for the research project. By recruiting 2 test centers geographically separated from one another and with different staff teams, we ensure a broader spectrum of HCWs performing the tests, improving the generalizability of our study results.

### Limitations

As we will enroll participants from public COVID-19 test centers during a mass-testing strategy, many of our participants will be tested for screening and not diagnostic purposes. Therefore, our study population will not be the same as the typical patients requiring a SARS-CoV-2 for diagnostic reasons in a future postpandemic period. However, in our study, we will both include participants being tested for diagnostic and screening reasons and we will, therefore, also have a unique insight into the virus detection during the asymptotic infectious phase.

### Conclusions

In conclusion, this study will be the first study to explore the role of collecting a palatine tonsil specimen in throat swabbing for SARS-CoV-2 molecular testing. As SARS-CoV-2 continues to infect people around the globe it seems relevant to have a deeper understanding of how to improve testing for this disease. Furthermore, the generalizability of performing a throat swab to test for upper respiratory virus may mean that the results of this study can be applied in test settings regarding other upper respiratory viruses.

## Appendix

Multimedia Appendix 1 Training and competency-based assessment of the skills of health care workers.

## Abbreviations

Ct: cycle threshold
GEE: generalized estimating equation
HCW: health care worker
NRS: numerical rating scale
RT-PCR: reverse transcriptase–polymerase chain reaction

## Data Availability

The data generated during this study are not publicly available but are available from the corresponding author upon reasonable request.
