# Supplementary material for: Molecular Detection of SARS-CoV-2 From Throat Swabs Performed With or Without Specimen Collection From the Tonsils: Protocol for a Multicenter Randomized Controlled Trial
Source: JMIR Res Protoc. 2024 Jun 12;13:e47446. doi: 10.2196/47446 (PMC11208824; doi:10.2196/47446)
Supplement: Multimedia Appendix 1 [file resprot_v13i1e47446_app1.docx]

Index

[A. Quality assurance checklists 2](#_Toc156373034)

[B. Survey for participants 4](#_Toc156373035)

[C. REDCap database 6](#_Toc156373036)

[D. Participant information 10](#_Toc156373037)

[E. Mallampati score 13](#_Toc156373038)

[F. Statistical Analysis Plan (SAP) 14](#_Toc156373039)

[1. Statistical principles 14](#_Toc156373040)

[1.1 Confidence intervals and P values 14](#_Toc156373041)

[1.2 Adherence and Protocol deviations 14](#_Toc156373042)

[2. Trial Population 15](#_Toc156373043)

[2.1 Screening data 15](#_Toc156373044)

[2.2 Eligibility 15](#_Toc156373045)

[2.3 Recruitment 15](#_Toc156373046)

[2.4 Withdrawal/Follow-up 16](#_Toc156373047)

[2.5 Baseline patient characteristics 16](#_Toc156373048)

[3. Analysis 17](#_Toc156373049)

[3.1 Outcome definitions 17](#_Toc156373050)

[3.2 Analysis methods 17](#_Toc156373051)

[3.3 Missing data 19](#_Toc156373052)

[3.4 Harms 19](#_Toc156373053)

[3.5 Statistical software 19](#_Toc156373054)

[G. Consent form 20](#_Toc156373055)

# A. Quality assurance checklists

Quality assurance checklist for assessing the skill level of health care workers participating in data collection in this randomized controlled trial investigating the diagnostic accuracy of throat swabs for SARS-CoV-2 testing performed with or without specimen collection from the palatine tonsils.

| **Checklist for oropharyngeal swab (OPS) including palatine tonsils** | | | | | | | | |
| --- | --- | --- | --- | --- | --- | --- | --- | --- |
| Name: | | Correct | | Incorrect | | | Not relevant | |
| 1 | OPS performed with proper use of protection equipment |  | |  | | |  | |
| 2 | OPS performed in compliance with infection prevention guidelines |  | |  | | |  | |
| 3 | OPS performed in alignment with the participant ensuring proper visualization of the back wall of the oropharynx |  | |  | | |  | |
| 4 | The swab is placed correctly between thumb, index and middle finger |  | |  | | |  | |
| 5 | Participant is instructed to say “aaaah” making the soft palate rises and a spatula is used |  | |  | | |  | |
| 6 | The swab is inserted and retracted without making contact with the mucous membrane of the oral cavity or the tongue |  | |  | | |  | |
| 7 | The swab gathers sufficient material from the back wall of the oropharynx including both palatine tonsils in a rotating or brushing movement |  | |  | | |  | |
| 8 | The swab is placed in the sample tube in accordance with local guidelines |  | |  | | |  | |
| Total score | |  | | | | | | |
|  | | Poor | Unacceptable | | Good | Acceptable | | Excellent |
| General assessment | |  |  | |  |  | |  |

| **Checklist for oropharyngeal swab (OPS) excluding palatine tonsils** | | | | | | | | |
| --- | --- | --- | --- | --- | --- | --- | --- | --- |
| Name: | | Correct | | Incorrect | | | Not relevant | |
| 1 | OPS performed with proper use of protection equipment |  | |  | | |  | |
| 2 | OPS performed in compliance with infection prevention guidelines |  | |  | | |  | |
| 3 | OPS performed in alignment with the participant ensuring proper visualization of the back wall of the oropharynx |  | |  | | |  | |
| 4 | The swab is placed correctly between thumb, index and middle finger |  | |  | | |  | |
| 5 | Participant is instructed to say “aaaah” making the soft palate rises and a spatula is used |  | |  | | |  | |
| 6 | The swab is inserted and retracted without making contact with the mucous membrane of the oral cavity or the tongue |  | |  | | |  | |
| 7 | The swab gathers sufficient material from the back wall of the oropharynx in a rotating or brushing movement |  | |  | | |  | |
| 8 | The swab is placed in the sample tube in accordance with local guidelines |  | |  | | |  | |
| Total score | |  | | | | | | |
|  | | Poor | Unacceptable | | Good | Acceptable | | Excellent |
| General assessment | |  |  | |  |  | |  |

# B. Survey for participants

Baseline survey for participants in this study investigating the diagnostic accuracy of throat swabs for SARS-CoV-2 testing performed with or without specimen collection from the palatine tonsils.

**Survey to be filled out by participant**

1. **Are you vaccinated against COVID-19?**

□YES □NO

1. **Have you previously been infected with COVID-19?**

□YES                         □NO

3. **Please indicate your reason for getting tested today:**

□Symptoms □Close contact □Positive quick test

□Screening □Test ahead of social gathering or nursing home visit   □Other

*If ’yes’ to symptoms, which symptoms do you have?*

□Sore throat   □Headache □Cough     □Muscle and joint pain

□Fever □Fatigue □Reduced sense of smell or taste

*If “yes” to symptoms, for how many days have you had symptoms?*

Indicate number of days: _____________day(s)

1. **Have you had your tonsils removed??**

□YES □NO

5. **On a scale from 0-10, how uncomfortable was today’s test for COVID-19?**

 No discomfort Worst possible discomfort


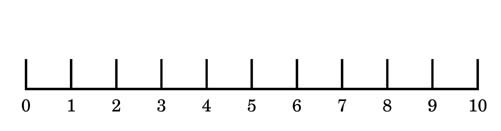


**_______________________________________________________________________________________**

**To be filled out by personnel:**

REDCap ID:________________________

Personnel ID:__________________________

Participant’s Mallampati score:

□**1** □**2** □**3** □**4**

# C. REDCap database

Outline of the REDCap database used to register baseline and follow up data of this randomized controlled trial investigating the diagnostic accuracy of throat swabs for SARS-CoV-2 testing performed with or without specimen collection from the palatine tonsils.

| REDCap-ID |  |
| --- | --- |
| Date | DD-MM-YYYY |
| Randomization | Oropharyngeal swab including palatine tonsils  Oropharyngeal swab excluding palatine tonsils |
| CPR-number |  |
| Sample ID |  |
| Are you vaccinated against COVID-19? | YES  NO |
| Have you previously been infected with COVID-19? | YES  NO |
| Please indicate your reason your reason for getting tested today | Symptoms  Close contact  Positive quick test  Screening  Test ahead of social gathering / visit to a nursing home  Other |
| If “YES” to symptoms, what symptoms do you have? | Sore throat  Headache  Cough  Muscle and joint pain  Fever  Fatigue  Reduced sense of smell or taste |
| If “YES” to symptoms, for how many days have you had symptoms? |  |
| Have you had your tonsils removed? | YES  NO |
| On a scale from 0-10, how uncomfortable was today’s test for COVID-19? | 0  1  2  3  4  5  6  7  8  9  10 |
| Personnel ID |  |
| Participant’s Mallampati score | 1  2  3  4 |
| FOLLOW UP | |
| What was your test result when you entered this study? | Positive  Negative |
| Have you been tested positive since entering the study? | YES  NO |
| Have you at any point since becoming infected and until four weeks after the test for Covid-19 had symptoms of Covid-19? | YES  NO |
| Did you experience any of the following symptoms after the test for Covid-19? | Sore throat  Headache  Cough  Muscle- / joint pain  Fever  Fatigue  Reduced sense of smell  Reduced sense of taste  Other |
| For how long after the test did you experience symptoms? | 1-3 days  3-5 days  5-7 days  1-2 weeks  2-3 weeks  3-4 weeks  More than 4 weeks |
| In total, how many days have you had symptoms of Covid-19 during the entire period you have been infected/symptomatic? |  |
| Do you currently have any symptoms or chronic signs of Covid-19? | YES  NO |
| Which of the following symptoms are you still experiencing? | Sore throat  Headache  Cough  Muscle- / joint pain  Fever  Fatigue  Reduced sense of smell  Reduced sense of taste  Other |
| Think about period from one week before your test until four weeks after.  Which of the following statements describes best how you felt at your worst? | I had no symptoms  I had symptoms but I was not too sick to go to work/school/other  I was too sick to go about my everyday life  I was admitted to the hospital due to COVID-19 |
| Have you taken days off work / school during your infection with COVID-19? | No, I have not taken any days off  Yes, I have taken days off during my infection with Covid-19  I am not employed |
| If ‘yes’ to having taken days off, how many days have you taken off in total? |  |
| Do you smoke? | Yes  No  I used to smoke, but I do not smoke anymore  Not relevant / I do not wish to disclose |
| If ‘yes’ to smoking, which of the following statements describes you best? | I smoke sometimes, e.g., at parties  I smoke daily (less than 10 cigarettes or equivalent to that)  I smoke daily (10 or more cigarettes or equivalent to that)  I smoke e-cigarettes  I do not know / I do not wish to disclose |
| Have you been diagnosed with a chronic illness by a doctor previous to your test for COVID-19? | No, I have not been diagnosed with a chronic illness  Yes, diabetes  Yes, asthma  Yes, COPD or chronic pulmonary disease  Yes, high blood pressure  Yes, chronic of frequent headache, including migraine  Yes, cancer  Other chronic illnesses |

# D. Participant information

Copy of the participant information given to all potential participants of the study in order to obtain informed consent to participation in this randomized controlled trial investigating the diagnostic accuracy of throat swabs for SARS-CoV-2 testing performed with or without specimen collection from the palatine tonsils. The information is in Danish.

**Deltagerinformation om deltagelse i videnskabeligt forsøg**

**Forsøgets titel**: Sammenligning af COVID-19 test ved podning i mundsvælget med eller uden mandler.

Vi vil spørge, om du vil deltage i et videnskabeligt forsøg, der udføres af Rigshospitalet i samarbejde med akutberedskabet i Region Hovedstaden og Hvidovre Hospital.

Før du beslutter, om du vil deltage i forsøget, skal du fuldt ud forstå, hvad forsøget går ud på, og hvorfor vi gennemfører forsøget. Vi vil derfor bede dig om at læse denne deltagerinformation. Du vil også modtage mundtlig deltagerinformation, hvor du kan stille de spørgsmål, du eventuelt har til forsøget.

Hvis du beslutter dig for at deltage i forsøget, vil vi bede dig om at underskrive en samtykkeerklæring. Det er frivilligt at deltage i forsøget, og du kan når som helst og uden at give en grund trække dit samtykke tilbage.

**Nytte ved forsøget**

Ved at indgå i forsøget hjælper du med at skaffe værdifuld viden om, hvordan man bedst foretager COVID-19 test. Det er med til at sikre, at vi kan bruge de testmetoder, der er bedst til at finde de smittede og samtidigt er mindst mulig ubehagelig for borgerne.

**Formål med forsøget**

Formålet med forsøget er at sammenligne den diagnostiske sikkerhed for to forskellige måder at foretage COVID-19 test på.

Normalt bliver man i Danmark podet med vatpind gennem munden for at samle materiale til PCR-test for COVID-19. Det varierer fra region til region hvorvidt podningen indbefatter mandlerne. Der mangler undersøgelser, der giver os viden om podning af mandlerne øger sikkerheden af testen eller ej. Vi vil derfor i dette lodtrækningsforsøg foretage podningen i svælget med eller uden samtidig podning af mandlerne for at undersøge, hvilken metode er den bedste. For at få viden om borger præferencer vil du også blive spurgt ind til testubehag ved undersøgelsen. Når studiet er overstået, vil vi således være klogere på hvilken metode der bør anbefales i danske test sammenhæng.

**Plan for forsøget**

Alle undersøgelser vil blive udført under samme besøg, og du kan forvente at dette kan tage omkring 5 minutter længere. Du vil få taget podning i munden samt besvare et kort spørgeskema.

Podningen i mundsvælget foregår ved, at man fører en vatpind ind bag ganen (standardpraksis). Bliver du randomiseret til at skulle have podet mandlerne også, foregår det ved samme podning. Prøven vil blive sendt til PCR-analyse som vanligt og svartiden vil ikke blive forlænget.

For at undersøge om testen også giver et resultatet som er i overensstemmelse med efterfølgende sygdomsudviklingen, vil vi eventuelt efter podningen også lave et opslag i din elektroniske patientjournal for at hente information om behov for indlæggelse, behov for medicinsk behandling, samt tidligere vaccinationsstatus og COVID-19 infektion. Ved positiv test vil du også blive kontaktet nogle uger efter afslutning af forsøget (e-boks eller telefon) for opfølgning på udviklingen af mulige alvorlige symptomer på COVID-19.

**Bivirkninger, risici, og ulemper**

Du kan muligvis forvente lidt ekstra ubehag ved podning af mandlerne, men ellers udføres podningen ligesom den aktuelle standard praksis.

**Etisk og databehandling (fortrolighed)**

Forsøget varetages på initiativ af Rigshospitalet, Region Hovedstadens Akutberedskab, og Hvidovre Hospital med uafhængig økonomisk støtte af Novo Nordisk Fonden. Projektet er godkendt af Videnskabsetisk Komite og Videnscenter for Dataanmeldelser, og håndtering af persondata vil blive håndteret med fuld fortrolighed efter gældende retningslinjer.

**Adgang til forsøgsresultater**

Forsøgets resultater vil blive offentliggjort umiddelbart efter forsøgets afslutning i et fagfællebedømt videnskabeligt tidsskrift.

Vi håber, at du med denne information har fået tilstrækkeligt indblik i, hvad det vil sige at deltage i forsøget, og at du føler dig rustet til at tage beslutningen om din eventuelle deltagelse. Hvis du har spørgsmål eller vil vide mere om forsøget, er du meget velkommen til at kontakte forsøgsansvarlig medicinstuderende Benedikte Hartvigsen (benedikte.hartvigsen@regionh.dk).

Vi håber du vil have mulighed for at bruge 5 minutter ekstra på at indgå i projektet i forbindelse med, at du skal testes for COVID-19.

På forhånd tak for din interesse i at hjælpe med vores forskningsprojekt!

På vegne af forskningsgruppen

Benedikte Hartvigsen, medicinstuderende, Københavns Universitet

Thomas Benfield, overlæge, professor, Infektionsmedicinsk Afdeling – Hvidovre Hospital

Annette Kjær Ersbøll, Professor, Region Hovedstadens Akutberedskab

Nina Steenhard, Ph.d., Testcenter Danmark – Statens Serum Institut

Tobias Todsen, speciallæge, lektor, Afdeling for Øre-Næse-Halskirurgi og Audiologi – Rigshospitalet

# E. Mallampati score

Depiction of the Mallampati score used by health care workers participating in data collection when assessing the participants in the study. The information is in Danish.


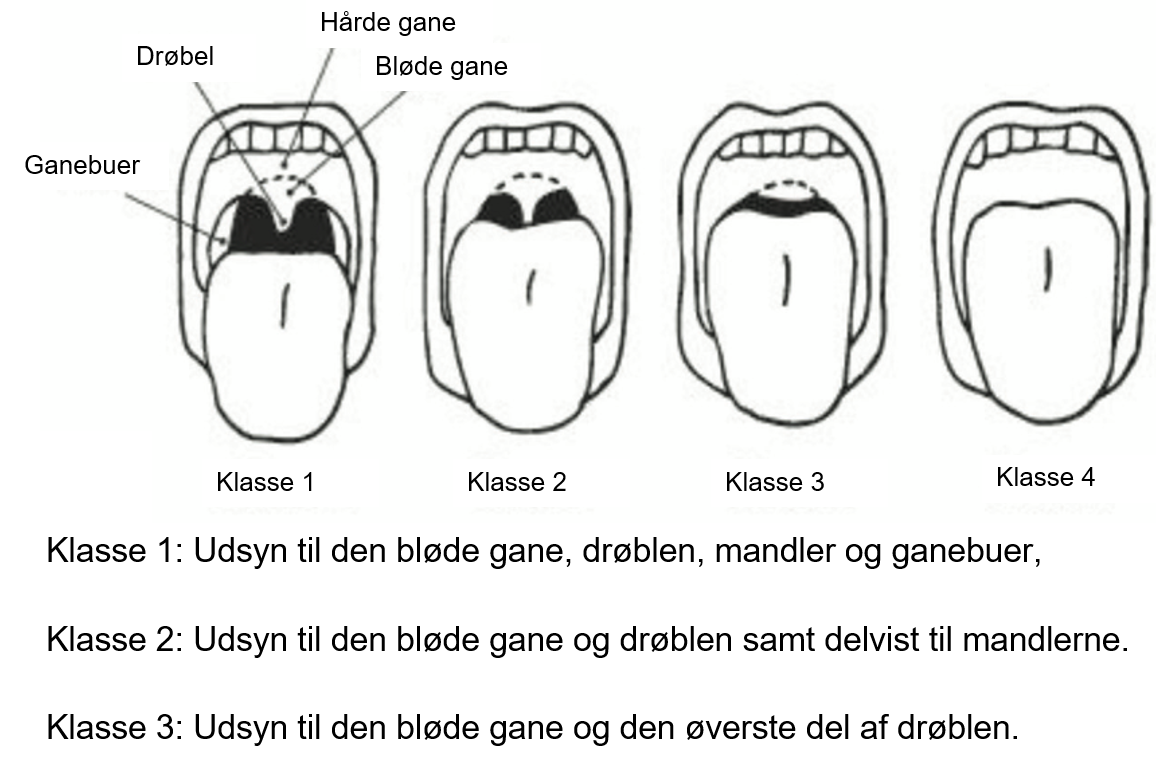


Klasse 4: Drøblen er ikke synlig overhovedet

# F. Statistical Analysis Plan (SAP)

Title: Molecular detection of SARS-CoV-2 from oropharyngeal swabs performed with or without specimen collection from the palatine tonsils – a multicenter randomized controlled trial

SAP version 1, 11122022

**Senior statistician**: Professor Annette Kjær Ersbøll, PhD

**Chief investigator**: Associate Professor Tobias Todsen, MD, PhD

## 1. Statistical principles

### 1.1 Confidence intervals and *P* values

The level of statistical significance will be p < 0.05 and 95% confidence interval will be reported.

### 1.2 Adherence and Protocol deviations

**Definition of adherence to the intervention**

Adherence is defined as participants who has a full registration of identification number (CPR number), test center site for collection of specimen and randomization to control or intervention group. Further, the participants need to complete the OPS specimen collection send for molecular testing.

Compliance is assessed based on the number and percent of subjects who have correct registration information and representative samples for RT-PCR.

**Description of adherence**

The adherence to the intervention will be summarized in the study flowchart and number and “% compliance” will be summarized.

**Definition of protocol deviations for the trial**

The participants will be excluded from final analysis if one or more of the following deviations from the testing protocol was fund:

Missing identification number (CPR number), no registered test center sites or no randomization (intervention or control) registered.

Missing RT-PCR result

**Description of which protocol deviations will be summarized**

The number and type of protocol deviation will be registered, and number of participants removed will be summarized in figure with study flow.

## 2. Trial Population

### 2.1 Screening data

We aim to invite individuals from Valby and Hillerød Covid-19 test centers to represent citizens from two urban areas in Copenhagen, Denmark to participate in the study.

### 2.2 Eligibility

All individuals being 18 years or older will be invited to participate in the SAMPLE trial. The same individual will only be allowed to participate in the study once.

The exclusion criteria were individuals with a tracheostomy, laryngectomy, or prior oropharyngeal cancer surgery without a Danish civil registration number (CPR).

### 2.3 Recruitment

A trial flow diagram will be used to summarize the number of included participants with information about:

- - Total number of Covid-19 tested individuals during the study period
  - Number of participants lost to identify / registrar
  - Number of participants excluded from final analyses due missing test results

### 2.4 Withdrawal/Follow-up

The level of withdrawal and the missing final RT-PCR test results during the study will be tabulated. As they only had a single OPS performed, no dropouts will be expected after the intervention – beside missing data.

### 2.5 Baseline patient characteristics

List of baseline characteristics for participants:

| Measure | Outcome | Description |
| --- | --- | --- |
| Demographic data | Age and gender | Data from the Danish civil registration number |
| Questionnaire | Test reason, symptom description, vaccination status, prior SARS-CoV-2 infection, prior tonsillectomy | Questionnaire registered in RedCap |
| NRS-scale | Discomfort score for OPS specimen collections | 11-item measure of test-related discomfort answered immediately after testing |
| Mallampati score | A visual assessment of the distance from the tongue base to the roof of the mouth of the participants | 4-item measure of amount of space in the mouth to reach the oropharynx assessed by the healthcare workers |

Categorical data will be summarized with number and percentage while continuous data will be summarized by mean and standard deviation. We will not perform tests of statistical significance for baseline characteristics.

## 3. Analysis

### 3.1 Outcome definitions

The primary outcome:

- - The proportion of positive SARS-CoV-2 RNA samples by RT-PCR for intervention and control group

The secondary outcome:

- - SARS-CoV-2 RT-PCR cycle threshold (Ct) value
  - Test discomfort on a 11-point NRS-scale
  - Development of COVID-19 disease after testing
  - SARS-CoV-2 detection rate for each healthcare worker
  - Mallampati Score of participants being tested

### 3.2 Analysis methods

**Analysis method and treatment effects**

Differences in the proportion of SARS-CoV-2 positive tests between the intervention and the control group will be compared using binary logistic regression using test center as fixed effect and a generalized estimating equation to adjust for clustering of data within the healthcare workers performing the sample. The difference in SARS-CoV-2 detection rate between healthcare workers will also be reported separately to estimate the inter-person variance. The Ct values from positive RT–PCR samples and the NRS discomfort scores will be compared using a general linear model with mixed effects (Ct) and GEE models (NRS discomfort).

The 95% confidence intervals (CI) will be presented. The level of statistical significance will be defined as p < 0.05.

**Adjustment for covariates**

The regression analyses will be adjusted for the effect of the test centers and the individual health-care worker performing the sample.

**Methods used for assumptions to be checked for statistical methods**

Assumptions for the logistic regression analysis included a binary outcome, independent observations and linearity in logit for continuous variables. To account for lack of independent observations, a generalized estimating equation approach will be applied. No continuous variables will be included.

Assumptions for the linear regression analysis included a normal distribution, independent observations, equal variation (homoscedasticity) and linearity in logit for continuous variables. Normally distributed outcomes and homoscedasticity will be evaluated visually by plots of the residuals. To account for lack of independent observations, a generalized estimating equation approach and mixed effect models will be applied. No continuous variables will be included.

**Details of alternative methods to be used if distributional assumptions do not hold, e.g., normality, proportional hazards, etc.**

If the assumption of a normal distribution of the outcome in the linear regression model is not fulfilled, a transformation of the outcome will be applied (e.g., logarithmic and rank transformations).

**Planned subgroup analyses**

We planned to do a sensitivity analysis using a lower cycle threshold (Ct) < 25 for positive SARS-CoV-2 definition to explore the consequences of a higher test specificity for the SARS-CoV-2 detection rate between specimen types. We also plan to estimate the sensitivity and specificity using Bayesian latent class analysis for accounting for an imperfect reference standard. Further, we planned to do subgroup analyses exploring the distribution of positive test results for participants stratified by symptoms, previous COVID-19 infection, vaccinations status, prior tonsillectomy and Mallampati score. To explore a potential bias from the distribution of the inconclusive test results, we excluded the inconclusive results in a subgroup analyses.

### 3.3 Missing data

Participants who will not adhere to the intervention definition (see SAP 1.2) will be reported as missing data and excluded from final analysis. Participants with missing data about their baseline characteristics from the questionnaire will still be included in the statistical analysis of primary outcome and secondary outcome. A table with baseline characteristics will be presented as raw data without the participants with missing data from the questionnaire.

### 3.4 Harms

Any adverse events during or after the collection of respiratory specimens for the trial will be noted and categorized into acute bleeding or foreign body in upper airway.

### 3.5 Statistical software

SAS statistical software suite ver. 9.4 (SAS Institute, North Carolina, U.S.)

# G. Consent form

Copy of the consent form signed by all participants after receiving oral and written informationa upon enrollment in the study. The information is in Danish.

**Informeret samtykke til deltagelse i et sundhedsvidenskabeligt forskningsprojekt**

Forskningsprojektets titel: Sammenligning af COVID-19 test ved podning af mundsvælget med eller uden mandler.

**Erklæring fra forsøgspersonen:**

Jeg har fået skriftlig og mundtlig information, og jeg ved nok om formål, metode, fordele og ulemper til at sige ja til at deltage.

Jeg ved, det er frivilligt at deltage, og at jeg altid kan trække mit samtykke tilbage uden at miste mine nuværende eller fremtidige rettigheder til behandling.

Jeg giver samtykke til at deltage i forskningsprojektet og har fået en kopi af den skriftlige information om projektet til eget brug.

Jeg samtykker i den forbindelse til, at mine testprøver for COVID-19 kan sendes til PCR-analyse på Statens Seruminstitut, og disse kan videregive oplysninger om resultatet til Region Hovedstaden til brug for den beskrevne undersøgelse. Jeg samtykker til, at der ved positivt testresultat kan gives tilladelse til at lave opslag i min elektroniske patientjournal, samt at jeg kan blive kontaktet (e-boks eller telefon) for opfølgning på udviklingen af mulige alvorlige symptomer på COVID-19.

Jeg har til enhver tid ret til at trække mit samtykke til videregivelserne tilbage ved kontakt til medicinstuderende Benedikte Hartvigsen via mail: benedikte.hartvigsen@regionh.dk.

Forsøgspersonens navn: _______________________________________

Telefonnummer: ____________________________________________

Underskrift:_________________________________________________
